# Fungicides alter the distribution and diversity of bacterial and fungal communities in ginseng fields

**DOI:** 10.1080/21655979.2021.1982277

**Published:** 2021-10-19

**Authors:** Guilong Ma, Xinxin Gao, Jie Nan, Tingting Zhang, Xiaobao Xie, Qi Cai

**Affiliations:** College of Plant Protection, Jilin Agricultural University, Changchun, China

**Keywords:** Fungicide, bacterial and fungal, diversity, abundance, ginseng field

## Abstract

The present study was focused on comparison of four typical fungicides in ginseng field to evaluate the impact of the different fungicides on the soil bacterial and fungal communities’ composition and diversity by using high-throughput sequencing. Five treatments were designed comprising carbendazim (D), dimethyl disulfide (E), dazomet (M), calcium cyanamide (S), and control (C). The application of fungicide obviously altered the distribution of dominant fungal and bacterial communities and remarkably decreased the diversity (1099-763 and 6457-2245). The most abundant *Proteobacteria* obviously degenerate in fungicide-treated soil and minimum in E (0.09%) compared to control (25.72%). The relative abundance of *Acidobacteria* was reduced from 27.76 (C) to 7.14% after applying fungicide and minimum in E. The phylum *Actinobacteria* are both decomposers of organic matter and enemies of soil-borne pathogens, elevated from 11.62 to 51.54% and are high in E. The fungi community mainly distributed into *Ascomycota* that enriched from 66.09 to 88.21% and highin M and E (88.21 and 85.10%), and *Basidiomycota* reduced from 21.13 to 3.23% and low in M and E (5.27 and 3.23%). Overall, environmentally related fungicides decreased the diversity and altered the composition of bacterial and fungal communities, highest sensitivity present in dimethyl disulfide-treated soil.

## Graphical abstract

**Figure:**
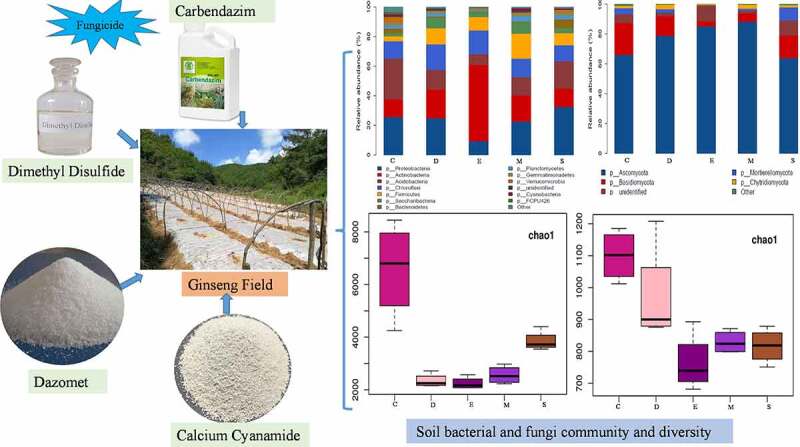

## 1. Introduction

Considering the ginseng (*Panax ginseng C. A. Meyer*) contributes as precious and high-value Chinese herbal medicine, while various microbes proliferate in soil during the long planting period, and once cross the soil tolerance limit, it will explode root rot or other diseases [1,2]. Therefore, it is very essential to the annual periodic maintenance of soil culture and disinfection. Fungicides are widely applied currently and still the most powerful weapon to prevent and control various pathogenic microorganisms during agrochemical crop protection industry [1]. The typical fungicide dazomet is a low-toxic and broad-spectrum soil fumigant, successfully replacing methyl bromide as well as widely used in ginseng fields to control plant pathogenic nematodes. Since dazomet could be decomposed in soil and generate toxic gases such as methyl isothiocyanate and formaldehyde that rapidly spreads to kill bacteria, fungi, nematodes, and weed seeds [3]. The fungicide dimethyl disulfide is derived from natural source and considered as an efficient nematicide to effectively control nematodes and soil-borne pathogens and weeds during high-value crop production [4]. At the same time, the fungicide carbendazim is widely used to control fungal pathogens or leaf diseases, and the fungicide calcium cyanamide is an effective ecological fertilizer for controlling soil-borne diseases and increasing nitrogen use efficiency. As calcium cyanamide can kill most root-knot nematodes and nematode eggs and generate toxic cyanamide liquid in soil by high temperature and solar radiation, thereby inhibiting the growth of root-knot nematodes, effectively protecting underground roots and producing nitrification inhibitor dicyandiamide during fungicide degradation process [5,6].

The microbial community as the vital core of soil ecological environment is responsible in nutrient cycling, plant growth, resistance, and stress response. There are many studies focuseing on fungicides that are responsible for microbial community composition and diversity as well as metabolic activity. Fang et al. [4] evaluated the efficiency of fungicides chloropicrin, dimethyl disulfide, dazomet, allyl isothiocyanate, and 1,3-dichloropropene on soil-borne diseases and pests’ control, and concluded the significant changes of denitrifying bacteria community abundance. Zhang et al. [7] and Han et al. [8] reported fungicide difenoconazole and tebuconazole obviously reduced bacterial community diversity and network complexity. Yan et al. [9] pointed out the presence of chloramphenicol increased the inhibitory effect of carbendazim on the fungi/bacteria ratio. Wang et al. [10] reported that the repeated application of carbendazim reduces soil microbial diversity and temporarily altered bacterial community composition, and suggest that carbendazim should be frequently applied during plant growing season to control fungal and foliage diseases of cultivable crops. The fungicides may cause an increase in Gram-positive bacteria and decrease in Gram-negative bacteria and fungi, and the beneficial microorganisms have the potential to prevent further infection of pathogen infection and control pests/diseases and promote plant growth, such as *Chaetomium, Pseudomonas,* and *Bacillus* that are considered as bio-controller and direct-attack soil-borne diseases [2,11,12].

The effect of fungicides on the soil microbial community varies with fungicides character, type, and dosage as well as soil and crop types. Accordingly, the present study focuses on the comparison of four typical fungicides dazomet, dimethyl disulfide, calcium cyanamide, and carbendazim in ginseng field to evaluate the impact of the different fungicides on the soil bacterial and fungal communities’ composition and diversity at the molecular level by using high-throughput sequencing.

## 2. Materials and methods

### 2.1. Experiment design and sample collection

The present study was carried out in ginseng field of Ji’an City (Jilin, China). Five treatments were designed and comprised 50% carbendazim wettable powder (D, provided by Fuda Agrochemical Co., Ltd., Jiangyin City, Jiangsu, China), 99.5% dimethyl disulfide agent (E, purchase from Baisite Reagent Co., Ltd, Chengdu, China), 98% dazomet particle agent (M, provided by Hisun Chemical Co., Ltd, Zhejiang, China), 50% calcium cyanamide granules (S, purchased from Zhaolong Science and Technology Development Co., Ltd, Jinan, China) and control (C, without any fungicide). Each treatment was performed in the divided block area (25 m^2^) with four times replication, the dosage of fungicide and active ingredient are listed in Table 1.

**Table 1.** The dosage of different fungicide and active ingredient in five treatments

Treatment	Fungicide	Dosage g/m^2^	Active Ingredient g/m^2^
C	-	-	-
D	50% Carbendazim wettable powder	5.0	2.5
E	99.5% Dimethyl disulfide agent	80.0	79.6
M	98% Dazomet particle agent	30.0	29.4
S	50% Calcium cyanamide granules	150.0	75.0

The soil samples (0–20 cm) of each plot were randomly collected according to the five-point method by stainless-steel cylindrical driller with a diameter of 5 cm and kept in a portable refrigerator (−20°C). After the soil samples were transported to the laboratory, passed through 2 mm sieve to remove plant tissues, roots, rocks, and stored at −20°C for microbial (bacterial and fungal) sequencing analysis.

### 2.2. Microbial diversity information analysis

Microbial sequencing was conducted by second-generation high-throughput sequencing technology to evaluate soil bacterial and fungal diversity information response of different fungicides. Firstly, total DNA was extracted according to Power Soil DNA Isolation Kit (MoBio Laboratories, Carlsbad, CA) instruction, and performed purity testing via Nano Drop 2000 UV-vis spectrophotometer (Thermo Scientific, Wilmington, USA). Then, target variable regions were amplified by PCR, use 338 F and 806 R primers (5-ACTCCTACGGGAGGCAGCAG-3 and 5-GGACTACHVGGGTATCTAAT-3) amplified V3-4 hypervariable region for bacterial, ITS1F and ITS2 primers (5-CTTGGTCATTTAGAGGAAGTAA-3 and 5-TGCGTTCTTCATCGATGC-3) amplified ITS region for fungal. For each soil sample, 8-digit barcode sequence was added to the 5 ends of the forward and reverse primers (provided by Allwegene Company, Beijing). The PCR was carried out by Mastercycler Gradient (Eppendorf, Germany) using 25 μL reaction volumes, containing 12.5 μL 2× Taq PCR Master Mix, 3 μL BSA (2ng/μL), 1 μL Forward Primer (5 μM), 1 μL Reverse Primer (5 μM), 2 μL template DNA, and 5.5 μL ddH_2_O. The cycling parameters were 95°C for 5 min, followed by 28 cycles of 95°C for 45 s, 55°C for 50 s and 72°C for 45s with a final extension at 72°C for 10 min [13]. The PCR products were purified by use of Agencourt AMPure XP nucleic acid purification kit. Followed step was Miseq library construction and performed high throughput sequencing on Miseq platform at Allwegene Company (Beijing, China).

### 2.3. Sequence process and data analyses

The raw sequences were first screened and quality controlled by Trimmomatic (v0.36) and Flash (v1.20) to remove low-quality score (≤20) and obtained fasta sequence, then remove chimera and short sequence to get high-quality sequence clean_tags. Then used Uparse algorithm of Vsearch (v2.7.1) clustered qualified clean tags into operational taxonomic units (OTUs) at 97% similarity [14]. The Ribosomal Database Project and BLAST Classifier tool were used to classify and annotate all sequences of different bacterial and fungal taxonomic levels according to SILVA128 and Unite database [15].

In order to compare bacterial and fungal communities in different samples, barplot diagram and heatmaps were generated based on taxonomic annotation by using R (v3.6.0) [16]. To evaluate the similarity between different fungicide-treated soil, alpha diversity index was done by QIIME (v1.8.0), and beta diversity-based principal co-ordinates and principal component analysis as well as nonmetric multidimensional scaling were performed by R (v3.6.0) [17]. Finally, the correlation among selected genes was performed in network by R (v3.6.0) igraph and psych package.

## 3. Results and discussion

### 3.1 Responses of dominant bacterial and fungal communities to different fungicides

**Figure 1.**
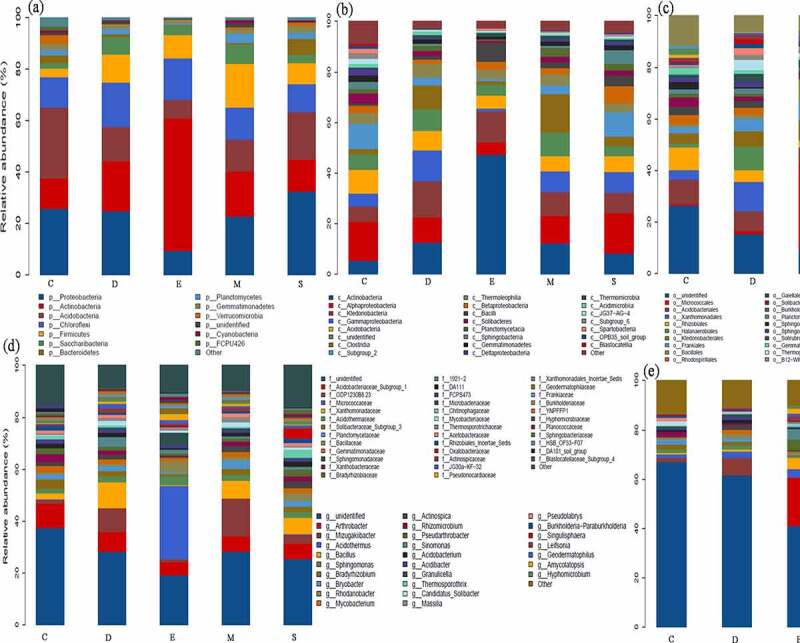
The dominant bacterial community response of different fungicides-based phylum (a), class (b), order (c), family (d), genus (e) level. the control (c), carbendazim (D), dimethyl disulfide (E), dazomet (m), and calcium cyanamide (s)

Among the samples, the dominant top five phyla were *Proteobacteria, Actinobacteria, Acidobacteria, Chloroflexi,* and *Firmicutes*. In particular, fungicide with calcium cyanamide (S) observed an obviously richness in *Proteobacteria* (32.39%), while decreased in carbendazim (24.88%) and dazomet (22.69%) treated soil, minimum in dimethyl disulfide (0.09%) compare with control (25.72%). The most abundant *Proteobacteria* was not surprising due to its high adaptability to unfavorable environments and dominant in carbon/nitrogen cycling [8,18]. The favorable habitat to *Proteobacteria* than *Acidobacterium* caused prevailing existence of *Pseudomonas* leading to adverse interference or disease on plant development [10,19]. Thus, the obvious degenerate tendency of *Proteobacteria* abundance after applying fungicides in this study revealed the positive effect of fungicide on beneficial microbial. Conversely, *Acidobacteria* could oxidize iron and deposit and caused surface rust in ginseng root [20], and the relative abundance (RA) of *Acidobacteria* was reduced from 27.76 to 7.14% and minimum in E. However, the phylum *Actinobacteria* was contributed as decomposers of organic matter and enemies of soil-borne pathogens or act as biocontrol agents to control plant diseases transmitted by soil and seeds [7,21]. The positive effect of fungicide demonstrated by elevated RA of *Actinobacteria* from 11.62 to 51.54% and significantly highin E (51.54%), followed by D (19.33%) and M (17.41%). Meanwhile *Chloroflexi* is related to organic halide respiration and increased the richness (11.82–17.33%) after applying fungicide. *Firmicutes* as the important actor during organic matter decomposition and carbon cycling, the RA increased (3.18–17.03%) in fungicide-treated soil [8]. More specifically, the class *Actinobacteria* was dominant in E (47.45%) while *Alphaproteobacteria* reduced from 15.29 to 4.84% and minimum in E. The abundance of *Ktedonobacteria* and *Gammaproteobacteria* were increased (6.09–1.09% and 5.11–12.32%) as well as richest in D-treated soil, and the richest genus of *Arthrobacter* and *Bacillus* were the highest in E (19.74% and 4.45%) (Figure 1). Similarly, Huang et al. [22] observed significantly increased in *Arthrobacter* abundance in dazomet-treated soil, which might be attributed by *Arthrobacter* ability to degrade dazomet as a nutrient source. And *Bacillus* is favorable to rhizobacteria that absorbs nutrients and promotes plant yield, and also potentially contributed as antimicrobial agents owing to the production of a variety of antibiotics, including bacitracin and lipopeptides, polyketones, and lipopeptides [23,24], further used in organic biological products to combat plant pathogens and inhibit various soil-borne pathogens.

**Figure 2.**
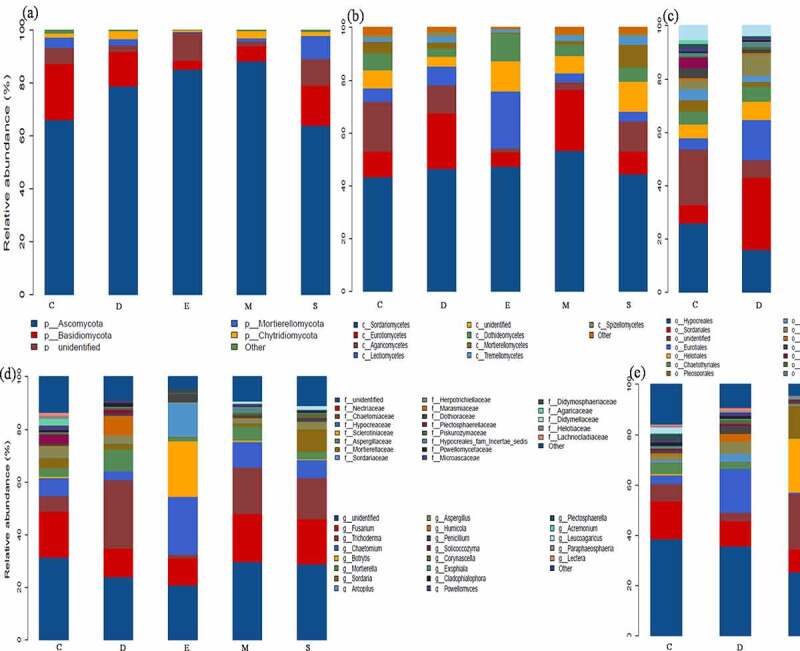
The dominant fungal community response of different fungicides-based phylum (a), class (b), order (c), family (d), genus (e) level. the control (C), carbendazim (D), dimethyl disulfide (E), dazomet (m), and calcium cyanamide (s)

In terms of fungi community, there are fewer types of dominant phyla compared to bacteria and mainly distributed into *Ascomycota* and *Basidiomycota* in fungicide-treated soil. The fungicide triggers *Ascomycota* enriched from 66.09 to 88.21% and high in M and E (88.21 and 85.10%), while *Basidiomycota* reduced from 21.13 to 3.23% and lowest in M and E (5.27 and 3.23%), meanwhile *Mortierellomycota* and *Chytridiomycota* account less than 1%. Particularly, the highest richness class *Sordariomycetes* was present in M (53.28%), and the RA of *Eurotiomycetes* increased from 9.51 to 21.11% and *Agaricomycetes* was reduced from 18.97 to 1.41%. Notably, the existence of dimethyl disulfide contributed competitive advantage for *Trichoderma* and *Botrytis* (21.82 and 21.15%) (Figure 2). The previous study also observed the predominant bacterial in ginseng soil is *Proteobacteria* and *Actinobacteria* and predominant fungi including *Ascomycota, Basidiomycota* and *Zygomycota* [2,12]. Han et al. pointed out that different fumigation obviously reduced the abundance of *Acidobacteria, Proteobacteria* and *Chloroflexi*, while increased *Actinobacteria, Firmicutes, Verrucomicrobia, Gemmatimonadetes,* and *Saccharibacteria* [8]. Overall, the application of fungicides regulated the dominant bacterial and fungal community’s abundance by promoting beneficial microbials.

### 3.2. The shift of dominant bacterial and fungal communities under different fungicides

**Figure 3.**
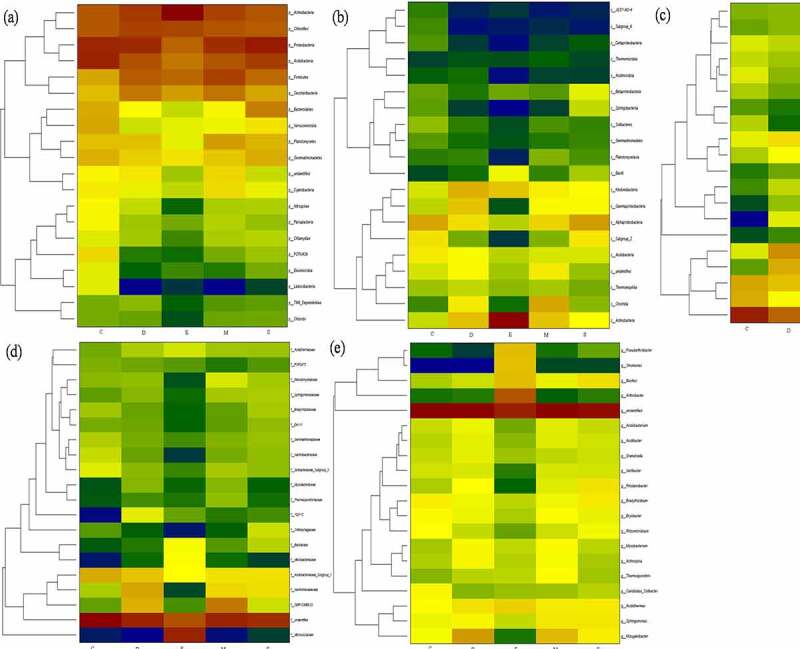
The shift of dominant bacterial community under different fungicides at phylum (a), class (b), order (c), family (d), genus (e) level. the control (C), carbendazim (D), dimethyl disulfide (E), dazomet (m), and calcium cyanamide (s)

**Figure 4.**
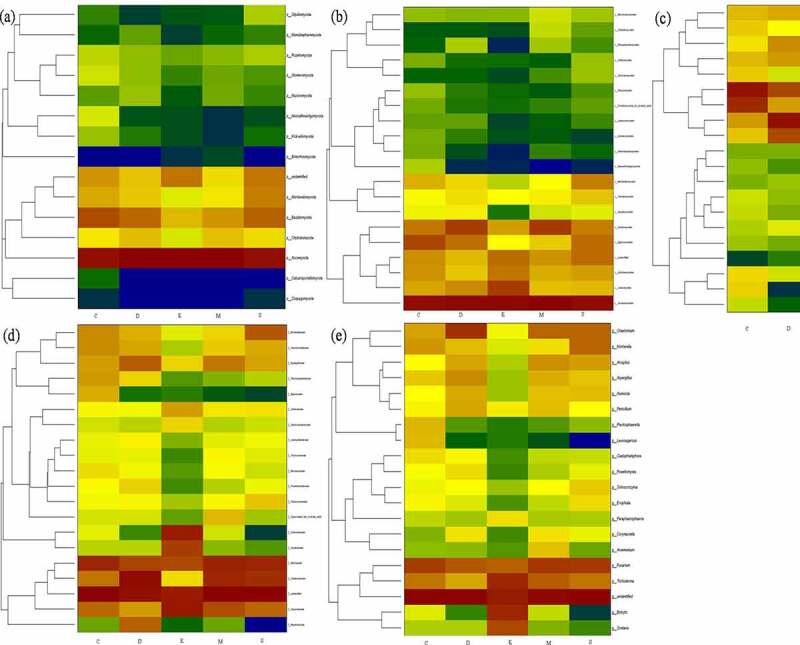
The shift of dominant fungal community under different fungicides at phylum (a), class (b), order (c), family (d), genus (e) level. the control (C), carbendazim (D), dimethyl disulfide (E), dazomet (m), and calcium cyanamide (s)

The application of fungicide is a remarkable shift of dominant bacterial and fungi communities. As shown the predominant microbial conversion from control to fungicide-treated soil in heatmap Figures 3 and 4. The richness bacterial *Proteobacteria* and *Acidobacteria* is converted into *Proteobacteria* and *Actinobacteria, Alphaproteobacteria* is converted in to *Actinobacteria* and *Clostridia, Acidobacteriales* and *Rhizobiales* are converted into *Micrococcales* and *Xanthomonadales*, and genus enriched in *Pseudarthrobacter, Sinomonas, Bacillus* and *Arthrobacter* after treated with fungicide. Additionally, the richness fungal from *Basidiomycota* is converted into *unidentified, Agaricomycetes* is converted into *Eurotiomycetes, Hypocreales* is converted into *Sordariales. Nectriaceae* is converted into *Hypocreaceae* and *Sclerotiniaceae*, and genus enriched in *Fusarium* and *Trichoderma* after treated with fungicide.

In particularly, the present study detected potential microbial for biocontrol and plant growth promotion, such as inhibition of disease-related bacteria *Bacillaceae, Streptomycetaceae, Micromonosporaceae, Chaetomium*, and *Bacillus*. While pathogenic fungal of *Cylindrocarpon, Alternaria, Rhizoctonia solani* and *Gibberella* were not identified in this study, it might be attributed by obligate parasitism. The *Actinomycete* members *Streptomyces* and *Micromonospora* can produce broad-spectrum of antibiotics, and enzymes further suppress multiple plant diseases by pathogen inhabitation [12,25]. However, *Fusarium* includes many members and mainly causes the pathogenicity of diverse plants (e.g., wheat, cucumber, peanut, and cotton) and related to agriculture, environment, and human health [2,26]. Baudy et al. [27] reported the root rot was caused by *Cylindrocarpon,* and *Fusarium* was the major reason for yield decreasing and poor growth of ginseng. And pathogenic potentialfungi *Mortierella* is widely distributed in temperate zone and usually colonized on plant roots and associated with replant disease [28]. Biofertilizer and biocontrol agent of *Chaetomium* could improve root mass and enzyme activity, and *Chaetomium globosum* can affect pathogen *Coniothyrium Diplodiella* and *Rhizopus stolonifera* [17,29]. Nicola et al. [28] found that *Bacillus* is effectively defensing against *Fusarium Wilt* and dominated in carbendazim treated soil.

The diverse dominant microbial phenomena were attributed by different types of fungicide affection. The application of dazomet obviously modified bacterial community by directly toxifying methyl isothiocyanate, and Eo et al. [30] identified the most resistant fungal to dazomet was *Trichoderma* and followed by *Zygorhynchus* and *Rhizopus*. Zhu et al. [31] noticed a rapidly increasing trend of *Penicillium, Nitrolancea,* and *Pseudomonas*, while decreasing tendency of *Fusarium* and *Mortierella, Nitrospira* and *Aquicella* after applying dazomet. Shen et al. [32] identified the dominant genus, *Bacteroides, Flavobacterium,* and *Cytophaga,* and the effect of carbendazim on bacterial community in arable land was mainly contributed by g-*Proteobacterium*. Fang et al. [33] reported that carbendazim alters bacterial community mostly owing to γ-*Proteobacteria*, and carbendazim may suppress *Pseudomonas, Mycobacterium, Bacillus, Arthrobacter, Burkholderia*, and *Streptococcus*. Additionally, dimethyl disulfide could be damaging the membrane of *Sclerotinia minor* and is responsible for antifungal activity, then induces host plants systemic resistance [34]. Previous studies have shown that the sensitivity of different microorganisms to cyanamide hydrogen is largely different, some nonpathogenic fungi including *Penicillium* and *Aspergillus* can degrade cellulose using cyanoamide as nitrogen source. The calcium cyanamide inhibits spore the formation and growth of pathogenic fungi based on intermediate metabolite hydrogen cyanide [13], and calcium cyanide non-targeting effect on microorganisms can be reflected by local microbial richness and diversity.

### 3.3. Alpha and beta diversity of bacterial and fungi communities after apply fungicide

**Figure 5.**
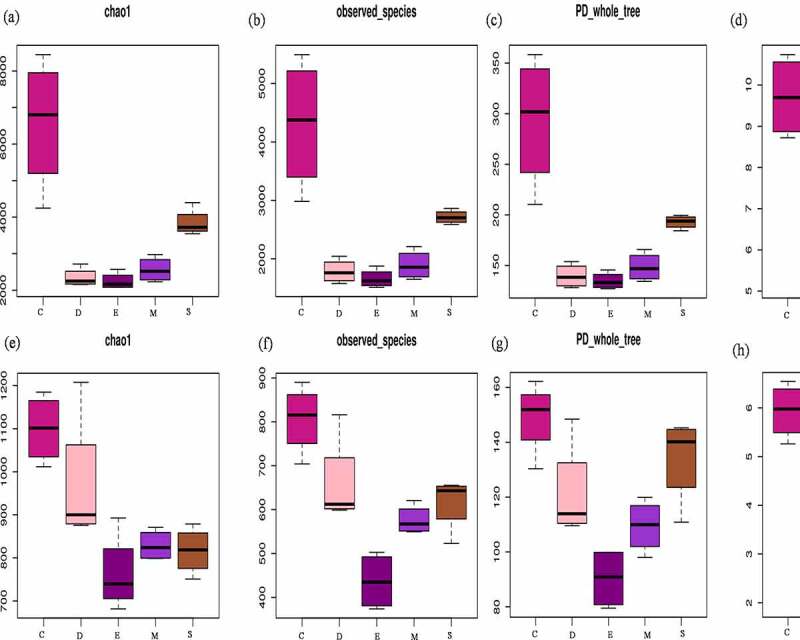
Alpha diversity of bacterial chao1(a), observed _species (b), PD whole tree (c) and shannon (d), and fungi chao1 (e), observed _species (f), PD whole tree (g) and shannon (h). the control (C), carbendazim (D), dimethyl disulfide (E), dazomet (m), and calcium cyanamide (s)

**Figure 6.**
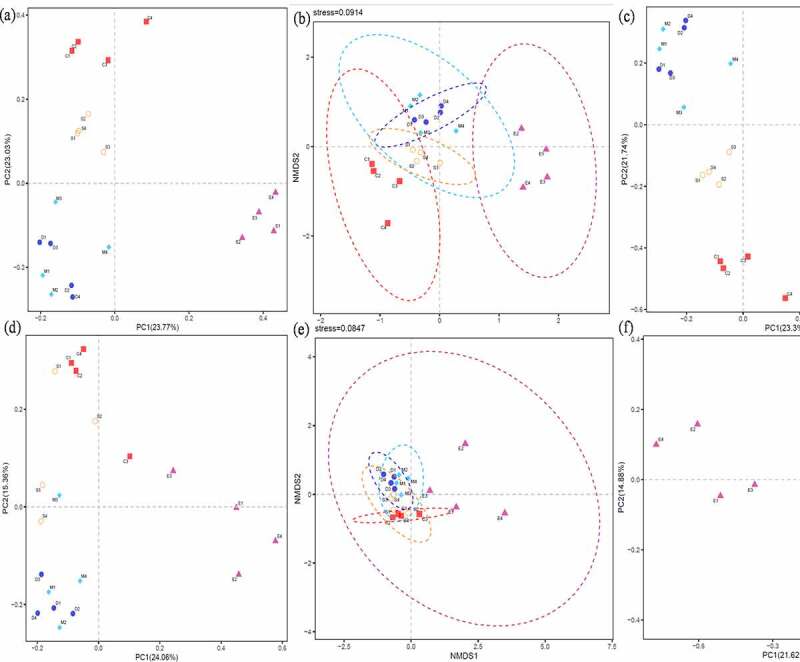
Beta diversity based bacterial principal co-ordinates (a), nonmetric multidimensional scaling (b), principal component analysis (c), fungal principal co-ordinates (d), nonmetric multidimensional scaling (e), principal component analysis (f). The control (C), carbendazim (D), dimethyl disulfide (E), dazomet (m), and calcium cyanamide (s)

The alpha and beta diversity of soil bacteria and fungi had significant changes in the different fungicide treatments (Figures 5 and 6). For bacterial, the richness index value of chao 1 decreased from 6457 in C to 2344, 2245, 2560, and 3846 in D, E, M, and S. The number of OTUs actually observed from 4306 (C) decreased to 1786, 1660, 1894, and 2714 in D, E, M, and S. Considering microbial abundance and the diversity index of evolutionary distance, PD whole tree decreased from 293 to 139, 134, 148, and 192 as well as Shannon index was 9.7, 8.3, 7.1, 8.4, and 8.9 in C, D, E, M, and S. On the other hand, about fungal community, chao 1 value decreased from 1099 (C) to 970, 763, 829, and 816 in D, E, M, and S. Observed species decreased from 806 (C) to 659, 436, 576, and 616 in D, E, M, and S. PD whole tree value was 149, 121, 90, 109, and 134 as well as Shannon was 5.9, 5.6, 3.1, 5.4, and 5.5 in C, D, E, M, and S. Therefore, fungicide application had strong interference on soil microbial community, bacterial, and fungal communities’ alpha-diversity remarkably decreased after treated with fungicide, consistent with [35]. Mumbi et al. [13] pointed out that the diversity and evenness of microorganism were decreased at the beginning of calcium cyanide treated soil, while soil bacteria are less sensitive to calcium cyanide than fungi. Zhang et al. and Nicola et al. [12,28] also reported bacterial diversity decreased significantly under carbendazim treated soil and bacteria have high tolerance to carbendazim. Tortella et al. [36] found out that carbendazim slightly influenced the amount of culturable fungi and bacteria, and carbendazim inhibited the functional diversity of bacterial communities in arable land, mainly due to its effect on g-*Proteobacterium*. In addition, Venn figure demonstrated that 1524 and 332 shared OTUs for bacterial and fungi, the unique OTUs 4972, 331, 173, 109, and 456 for bacterial while 257, 100, 41, 76, and 278 for fungi during C, D, E, M, and S (Figure S1). Therefore, soil respiration activities are mainly contributed by bacterial metabolism, and the respiration of bacteria is more sensitive and approximately three times than fungi, that is opposite to Bamaga et al. [11] who observed stronger fungal respiration than bacteria and pointed out the inhibition of carbendazim on soil fungi is greater than bacteria.

Similarly, Zhu et al. [31] reported that the Venn diagram presenting 1524 and 332, which overlapped OTUs for bacterial and fungi. Wang et al. [37] elaborated that soil bacterial community structure changed after apply carbendazim, and the inhibition of bacterial community by carbendazim is temporary. Furthermore, the beta diversity analysis during bacterial and fungal both identified that C and S were gathered one group, M and D were clustered into one unit, while E was individually distributed based on PCoA and PCA. While from perspective of NMDS analysis, E and C were distributed separately and remarkably dispersed bacterial/fungi community, while other three fungicides (M, D, and S) had weaker dispersion effect on bacterial community. Finally, the results of alpha and beta diversity demonstrated the bacteria and fungi community decreased significantly.

### 3.4. The correlation of predominant bacterial and fungal communities

**Figure 7.**
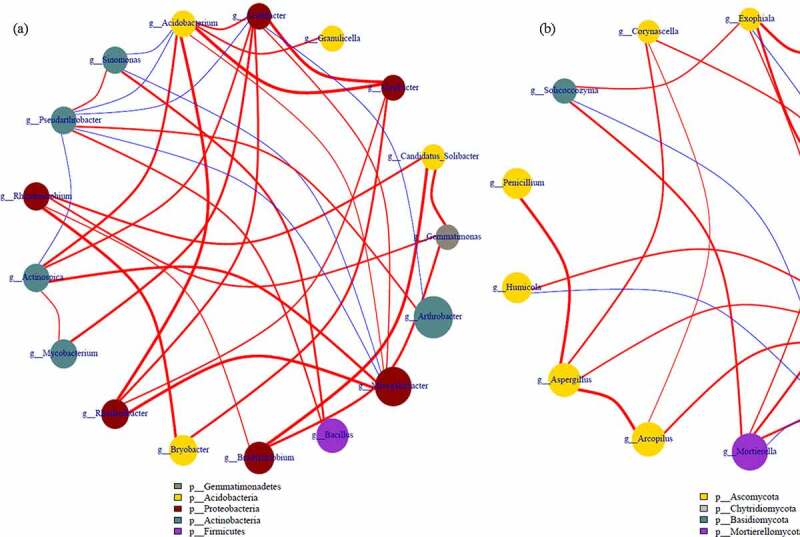
The correlation of selected bacterial (a) and fungal (b) communities-based network analysis

The correlation among top 20 bacterial and fungi communities in genus level is demonstrated in network Figure 7. The genus *Acidobacterium* was positive with *Variibacter* (0.61), *Granulicella* (0.74) and *Acidibacter* (0.73), while negative with *Gemmatimonas* (−0.10), *Candidatus_Solibacter* (−0.17) and *Thermosporothrix* (−0.01). The genus *Rhizomicrobium* was positive with *Gemmatimonas* (0.74) and *Candidatus_Solibacter* (0.69) while negative with *Sinomonas* (−0.55). The genus *Rhodanobacter* was positive with *Variibacter* (0.77) and *Acidibacter* (0.68), negative with *Sinomonas* (−0.46) and *Pseudarthrobacter* (−0.41). And *Pseudarthrobacter* was positive with *Sinomonas* (0.81) and negative with *Acidibacter* (−0.72) and *Acidobacterium* (−0.83). With regard to fungal, *Mortierella* was positive with *Exophiala* (0.82), *Lectera* (0.69), and *Solicoccozyma* (0.69). The genus *Chaetomium* was positive with *Corynascella* (0.78) and *Aspergillus* (0.77). The genus *Exophiala* was positive with *Lectera* (0.65) and *Powellomyces* (0.64), and negative with *Paraphaeosphaeria* (−0.12) and *Leucoagaricus* (−0.09). The genus *Solicoccozyma* was positive with *Exophiala* (0.80) and *Corynascella* (0.48). While genus *Botrytis* was negative with *Lectera* (−0.49), *Powellomyces* (−0.52), *Exophiala* (−0.80) *Solicoccozyma* (−0.74), *Humicola* (−0.72) and *Mortierella* (−0.79).

Accordingly, the correlation of bacterial communities was more complex than fungi, and fungicides reduced the complexity of microbial symbiosis network which is due to the reduction of functional microorganisms and consistent with Fournier et al. and Li et al. [38,39]. The main prevailence of *Proteobacteria* and *Ascomycota* in fungicide-treated soil indicated high resistance to chemical, similarly results are also found in the previous studies [40–43]. Therefore, environmentally related fungicides decreased the diversity, altered the composition and interaction of bacterial and fungal communities rather than function. Han et al. [8] conclude that the application of fungicides didn’t significantly alter the dominant fungi and bacteria communities, but the abundance changed obviously. Interestingly, the comparison of the microbial community treated with different fungicides showed that the RA of dominant beneficial microorganisms enriched and pathogen decreased, including bacterial of *Bacillus, Pseudomonas* and *Arthrobacter* fungi of *Fusarium, Mortierella* and *Botrytis*. Therefore, it is inferred that fungicide activates the defense activities of plants by changing the soil microbial community, and the adaptability and enrichment of beneficial microorganisms and reduction of pathogenic bacteria in soil may be the role of fungicides application.

## 4. Conclusion

Fungicide application strongly interfered on soil microbial community, remarkably decreased bacterial and fungal communities’ diversity. The respiration of bacteria is more sensitive and approximately three times than fungi. The abundance of dominant bacterial *Proteobacteria* decreased and fungal *Ascomycota* increased in fungicide treated soil, especially enriched in beneficial microorganisms and reduced in pathogen, including bacterial of *Bacillus, Pseudomonas* and *Arthrobacter*, fungi of *Fusarium, Mortierella* and *Botrytis*. The adaptability and enrichment of beneficial microorganisms and reduction of pathogenic bacteria in soil may be the role of fungicides application.

## Supplementary Material

Supplemental Material
