# Supplementary material for: Fungicides alter the distribution and diversity of bacterial and fungal communities in ginseng fields
Source: Bioengineered. 2021 Oct 19;12(1):8043–56. doi: 10.1080/21655979.2021.1982277 (PMC8806933; doi:10.1080/21655979.2021.1982277)
Supplement: Supplemental Material [file KBIE_A_1982277_SM2333.docx]

10- September-2021

To

**Prof. Mohammad Taherzadeh**

**Editor-in-Chief**

**Bioengineered**

Dear Prof. Mohammad,

I have attached the revised manuscript ‘***Fungicides alter the distribution and diversity of bacterial and fungal communities in ginseng fields****’* for the favor of review and application in Bioengineered.

The response to the comment is provided separately while the revisions made were highlighted in yellow color for the easy reference.

Thank you.

Yours sincerely,

**Prof. Guilong Ma,**

College of Plant Protection,

Jilin Agricultural University,

Changchun 130118, China

E-mail: guilongma@163.com

**Response to Editor/Reviewer’s Comments**

**Manuscript Number:** KBIE-2021-2600R1

**Manuscript title:** Fungicides alter the distribution and diversity of bacterial and fungal communities in ginseng fields

**Article Type:** Original research paper

Firstly, we are thankful to all the reviewers for their valuable time and effort given to review our manuscript. We sincerely appreciate all the valuable comments and suggestions that certainly helped us to improve the quality of the manuscript. The responses to the comments are provided below in point to point manner as raised by the reviewers. Appropriate revisions made were highlighted in yellow color for the easy reference. Additionally, other minor corrections/revisions were made in the manuscripts that have also been highlighted in yellow color. We are sure that this would satisfy reviewer’s concerns. Please find below the responses to the corrections raised by the reviewers, along with the list of changes that we have made in the revised manuscript. In addition to the changes suggested by the reviewers, we have also corrected some language and statistical errors in the manuscript.

**Reviewer 1:** "Responses of different fungicides to diversity reduction and abundance distribution of bacterial and fungal communities in ginseng field", authors have focuses on four typical fungicides (carbendazim, dimethyl disulfide, dazomet, and calcium cyanamide) in ginseng field to evaluated the soil bacterial and fungal communities' composition and diversity. The topic is valuable but in order to improve the quality, minor revision need to be done as the following suggestions:

**Response:** Thank you for your approval and comments for this study. It helps to improve the quality of this study.

**Comment 1:** Revised Line no. 70-71.

**Response:** Revised according to suggestion in line no. 44-46: dazomet (M), calcium cyanamide (S) and control (C). The application of fungicide obviously altered the dominant fungal and bacterial communities’ distribution and remarkably decreased the diversity (1099-763 and 6457-2245).

**Comment 2:**  Need clear mention, 27.76 is for control? s

**Response:** Thank you very much for suggestion, yes, 27.76 is for control, revised in line no. 49**:** The relative abundance of *Acidobacteria* was reduced from 27.76 (C) to 7.14% after apply fungicide and minimum in E.

**Comment 3:**  Need detailed description of fungicides decreased the diversity.

**Response:** As suggested, we revised in line no. 44-46: The application of fungicide obviously altered the dominant fungal and bacterial communities’ distribution and remarkably decreased the diversity (1099-763 and 6457-2245).

**Comment 4:** Line no. 87-91,95-100 split into two sentences for better understanding.

**Response:** Thank you very much for suggestion, we have revised the sentences in line no. 61-65: The typical fungicide dazomet is a low-toxic and broad-spectrum soil fumigant, successfully replaced methyl bromide as well as widely used in ginseng fields to control plant pathogenic nematodes. Since dazomet could be decomposed in soil and generate toxic gases such as methyl isothiocyanate and formaldehyde that rapidly spreads to kill bacteria, fungi, nematodes and weed seeds [2]; Line no. 68-75: At the same time, the fungicide carbendazim is widely used to control fungal pathogens or leaf diseases and the fungicide calcium cyanamide is an effective ecological fertilizer for controlling soil-borne diseases and increasing nitrogen use efficiency. Due to calcium cyanamide can kill most root-knot nematodes and nematode eggs and generate toxic cyanamide liquid in soil by high temperature and solar radiation, thereby inhibiting the growth of root-knot nematodes, effectively protecting underground roots and producing nitrification inhibitor dicyandiamide during fungicide degradation process [4-5].

**Comment 5:** Complete the information "The properties of the soil were:" in 2.1. Experimental design and sample collection Line no. 131.

**Response:** Thank you very much for suggestion, revised accordingly.

**Comment 6:** Line no.189-194 very difficult to understand, revise.

**Response:** We have revised the sentences in line no. 162-171: In particularly, fungicide with calcium cyanamide (S) observed an obviously richness in *Proteobacteria* (32.39%), while decreased in carbendazim (24.88%) and dazomet (22.69%) treated soil, minimum in dimethyl disulfide (0.09%) compare with control (25.72%). The most abundant *Proteobacteria* was not surprising due to its high adaptability to unfavorable environments and dominant in carbon/nitrogen cycling [7, 18]. The favorable habitat to *Proteobacteria* than *Acidobacterium* caused prevailing existence of *Pseudomonas* that leading to adverse interference or disease on plant development [19-20]. Thus, the obvious degenerate tendency of *Proteobacteria* abundance after apply fungicides in this study revealed the positive effect of fungicide on beneficial microbial.

**Comment 7:** Carefully check the format, such as Gammaproteobacteria, Sclerotinia minor should be Italic in Line no. 207 and 279.

**Response:** Thank you very much for suggestion, we have revised in line no. 184 and 254: *Gammaproteobacteria* and *Sclerotinia minor.*

**Comment 8:** What's the meaning of "stable" in Line no. 217.

**Response:** Thank you very much for comment, we have revised in line no. 194 “dominant”.

**Comment 9:** Information need complete in Line no. 237-244 ....convert into...after fungicide apply?

**Response:** Thank you very much for suggestion, we have completed in line no. 214-215: As shown the predominant microbial conversion from control to fungicide treated soil in heatmap Figure 3 and 4.

**Comment 10:** Line no. 346-348,351 need revise.

**Response:** Thank you very much for suggestion, revised in line no. 323-327: The main prevailed of *Proteobacteria* and *Ascomycota* in fungicide treated soil indicated high resistance to chemical, similarly result also found in previous study [41-44]. Therefore, environmentally related fungicides decreased the diversity, altered the composition and interaction of bacterial and fungal communities rather than function.

**Comment 11:** In the reference list, please check all the reference and revise according to the Journal guidelines.

**Response:** Yes. We have checked all references carefully.

**Reviewer 2:** Manuscript Number: KBIE-2021-2600

Full Title: Responses of different fungicides to diversity reduction and abundance distribution of bacterial and fungal communities in ginseng field

Article Type: Research Article

Comments:

**Comment 1:** Revise the title for better presentation;

**Response:** Thank you very much for suggestion, revised as: Fungicides alter the distribution and diversity of bacterial and fungal communities in ginseng fields.

**Comment 2:**    Revise the highlights for the better clarity;

**Response:** Thank you very much for suggestion, it has been revised and marked with yellow color.

Highlight

1. Comparative evaluated the bacterial and fungal communities under four typical fungicides.
2. The bacterial and fungal diversity decreased (6457-2245 and 1099-763) after apply fungicide.
3. The prevailed microbial was *Proteobacteria* (32.39-0.09%) and *Ascomycota* (66.09-88.21%).
4. The application of fungicides enriched beneficial microorganisms and decreased pathogen.

**Comment 3:** Add background information in the abstract first few sentences;

**Response:** Thank you very much for suggestion, the background of this study is considering the ginseng (*Panax ginseng C. A. Meyer*) contribute as precious and high-value Chinese herbal medicine, while various of microbes will proliferation in soil during the long planting period, and once cross the soil tolerance limit will explode root rot or other diseases. Therefore, it is very essential to annual periodic maintenance of soil culture and disinfection. Accordingly, we focus on comparation of four typical fungicides dazomet, dimethyl disulfide, calcium cyanamide and carbendazim in ginseng field to evaluated the impact of the different fungicides on the soil bacterial and fungal communities’ composition and diversity at molecular level by using high-throughput sequencing. But due to the limited length of the abstract, we didn’t add in abstract and present in introduction, please understand.

**Comment 4:** Cite most recent references in the introduction part;

**Response:** Thank you very much for the suggestion, please we make correction according to suggestion.

**Comment 5:** Revise the heading 2.1;

**Response:** Thank you very much for the suggestion, revised as per suggestion. 2.1. Experiment design and sample collection

**Comment 6:**  Format the manuscript as per journal author guideline, especially for results and discussion separate;

**Response:** Thank you very much for the suggestion, revised accordingly.

**Comment 7:**  Correct the references as per journal format;

**Response:** Thank you very much for the suggestion, corrected according to suggestion.

**Comment 8:**  Delete old references from discussion and cite most recent references for wide readers interest;

**Response:** Thank you very much for the suggestion, revised as per suggestion.

**Comment 9:**  Nano Drop spectrophotometer (Thermo Scientific): write the country name of used equipment;

**Response:** Thank you very much for the suggestion, revised as per suggestion: Nano Drop 2000 UV-vis spectrophotometer (Thermo Scientific, Wilmington, USA).

**Comment 10:**  Revise line no. 286.

**Response:** Thank you very much for the suggestion, revised as per suggestion in line no. 264-267: For bacterial, the richness index value of chao 1 decreased from 6457 in C to 2344, 2245, 2560 and 3846 in D, E, M and S. The number of OTUs actually observed from 4306 (C) decreased to 1786, 1660, 1894 and 2714 in D, E, M and S.

**Reviewer 3:** "Responses of different fungicides to diversity reduction and abundance distribution of bacterial and fungal communities in ginseng field." --The author provides a data-rich manuscript. Detailed results are presented in the manuscript. However, some conclusions should be revised before being accepted.

Specific suggestions:

**Comment 1:**  Highlights need to be resubmitted (maximum 85 characters, including spaces).

**Response:** Thank you very much for the suggestion, revised as per suggestion Highlight

1. Comparative evaluated the bacterial and fungal communities under four typical fungicides.
2. The bacterial and fungal diversity decreased (6457-2245 and 1099-763) after apply fungicide.
3. The prevailed microbial was *Proteobacteria* (32.39-0.09%) and *Ascomycota* (66.09-88.21%).
4. The application of fungicides enriched beneficial microorganisms and decreased pathogen.

**Comment 2:**  Abstracts should be concise and extract important innovations, not a lot of data.

**Response:** Thank you very much for the suggestion, revised as per suggestion: Present study was focuses on comparation of four typical fungicides in ginseng field to evaluated the impact of the different fungicides on the soil bacterial and fungal communities’ composition and diversity by using high-throughput sequencing. Five treatments were designed and comprise carbendazim (D), dimethyl disulfide (E), dazomet (M), calcium cyanamide (S) and control (C). The application of fungicide obviously altered the dominant fungal and bacterial communities’ distribution and remarkably decreased the diversity (1099-763 and 6457-2245). The most abundant *Proteobacteria* obvious degenerate in fungicides treated soil and minimum in E (0.09%) compare with control (25.72%). The relative abundance of *Acidobacteria* was reduced from 27.76 (C) to 7.14% after apply fungicide and minimum in E. The phylum *Actinobacteria* are both decomposers of organic matter and enemies of soil-borne pathogens, elevated from 11.62 to 51.54% and highest in E. The fungi community mainly distributed into *Ascomycota* that enriched from 66.09 to 88.21% and highest in M and E (88.21 and 85.10%), and *Basidiomycota* reduced from 21.13 to 3.23 % and lowest in M and E (5.27 and 3.23%). Overall, environmentally related fungicides decreased the diversity and altered the composition of bacterial and fungal communities, highest sensitivity present in dimethyl disulfide treated soil.

**Comment 3:**  I don't know why the author chose these four fungicides, although they were introduced in too much detail in the introduction，A typical? Different category or traditional?

**Response:** Thank you very much for the suggestion, yes, we focus on comparation of four typical and traditionally applied fungicides dazomet, dimethyl disulfide, calcium cyanamide and carbendazim in ginseng field to evaluated the impact of the different fungicides on the soil bacterial and fungal communities’ composition and diversity. Since considering the ginseng (*Panax ginseng C. A. Meyer*) contribute as precious and high-value Chinese herbal medicine, while various of microbes will proliferation in soil during the long planting period, and once cross the soil tolerance limit will explode root rot or other diseases. Therefore, it is very essential to annual periodic maintenance of soil culture and disinfection.

**Comment 4:**   Ginseng field should be mentioned more than simply soil.

**Response:** Thank you very much for the suggestion, revised in line no. 96-100: Considering the ginseng (*Panax ginseng C. A. Meyer*) contribute as precious and high-value Chinese herbal medicine, while various of microbes will proliferation in soil during the long planting period, and once cross the soil tolerance limit will explode root rot or other diseases [12]. Therefore, it is very essential to annual periodic maintenance of soil culture and disinfection.

**Comment 5:**  Letters need to be rotated 90 degrees on the x-coordinate of the Figure.

**Response:** Thank you very much for the suggestion, all figures have been completed.

**Comment 6:**  The diagram corresponding to the content discussed in the text should be informed first.

**Response:** Thank you for your comments, revised as per suggestion.

**Comment 7:** Page 12, line 189- It is true that P is most abundant in S, but Proteobacter is not most abundant in other disposal groups and is less than C. Why does the author mention "Due to its high adaptability to unfavorable environments and dominant in carbon/nitrogen cycling"?

**Response:** Thank you for your comments, we select the most dominant phylum Proteobacteria is according to the average value of relative abundance, since average abundance of Chloroflexi and Proteobacteria were 13.72% and 23% based all sample.

In particularly, fungicide with calcium cyanamide (S) observed an obviously richness in Proteobacteria (32.39%), while decreased in carbendazim (24.88%) and dazomet (22.69%) treated soil, minimum in dimethyl disulfide (0.09%) compare with control (25.72%). We mention due to its high adaptability to unfavorable environments and dominant in carbon/nitrogen cycling, aimed to explain why richest in Proteobacteria.

**Comment 8:**  There is more discussion at the phylum, but not at the Class, Order, Family, Genus, so useless parts should be removed from Figs. 1 and 2, or more discussion should be added for Figs. 1 and 2.

**Response:** Thank you for your comments, revised in line no. 181-186: More specifically, the class *Actinobacteria* was dominant in E (47.45%) while *Alphaproteobacteria* reduced from 15.29 to 4.84 % and minimum in E. The abundance of *Ktedonobacteria* and *Gammaproteobacteria* were increased (6.09-1.09% and 5.11-12.32%) as well as richest in D treated soil, and the richest genus of *Arthrobacter* and *Bacillus* were highest in E (19.74% and 4.45%) (Figure 1).

**Comment 9:** Too many other people's reports were introduced into the results and discussions, What is the author's aim? Support the author's view? However, no relevant treatise was found.

**Response:** Thank you for your comments, we revised accordingly, we mention other people's research during discussions, because of the relevance in terms of bacterial and fungal communities or the efficiency of different fungicides application, but due to the content length limitations, we didn’t detail summary and comparison to much. I hope you understand.

**Comment 10:** More mechanisms or reasons for change should be discussed rather than simply enumerating data.

**Response:** Thank you for suggestion, revised according to suggestion.

**Reviewer 4 (Editorials):**

**Comment 1:** In all your responses to all reviewers and editors, you should mention also “where” in your manuscript and how you did the revisions (use different font colors for the comments and your responses). Also, all revision in the manuscript should be visible using different font color or highlighted text. As alternative, you can provide your revised manuscript in two versions, one with clean text and also one version using track changes and submit both.

**Response:** Thank you for suggestion, we highlighted revised part with yellow pen, and provide two version.

**Comment 2:** While submitting the revised manuscript, please double check the “author names” and “affiliation address” provided in the submission so that authorship related changes are made in the revision stage. If your manuscript is accepted, any authorship change will involve approval from co-authors and respective editor handling the submission and this may cause a significant delay in publishing your manuscript.

**Response:** Thank you very much for the suggestion, we have checked carefully.

**Comment 3:** Provide research highlights. The highlights should be 3 to 5 bullet points maximum 85 characters, including spaces, per bullet point (practically in one line). Highlights should reflect the main findings. They should not describe, what was done. You may put the highlights in a separate word file or add to the manuscript after the keywords. The highlights and graphical abstract improve the visibility and citation of the work.

**Response:** Thank you for suggestion, please we have provided in revised manuscript.

Highlight

1. Comparative evaluated the bacterial and fungal communities under four typical fungicides.
2. The bacterial and fungal diversity decreased (6457-2245 and 1099-763) after apply fungicide.
3. The prevailed microbial was *Proteobacteria* (32.39-0.09%) and *Ascomycota* (66.09-88.21%).
4. The application of fungicides enriched beneficial microorganisms and decreased pathogen.

**Comment 4:**   Your manuscript needs a professional revision for the language. You may use any professional service to improve its language. If you prefer to use services offered by Bioengineered, you may refer to instruction for authors or www.tandfeditingservices.com.

**Response:** Thank you very much for the suggestion, revised as per suggestion.

**Comment 5:**  Add clearly the hypothesis, aims and goals of this work to the last paragraph to your introduction.

**Response:** It has been added and marked with yellow pen: Accordingly, present study focuses on comparation of four typical fungicides dazomet, dimethyl disulfide, calcium cyanamide and carbendazim in ginseng field to evaluated the impact of the different fungicides on the soil bacterial and fungal communities’ composition and diversity at molecular level by using high-throughput sequencing.

**Comment 6:**  All your methods should have appropriate citation of references. Check and revise them properly.

**Response:** Thank you very much for the suggestion, revised as per suggestion.

**Comment 7:** Please add a starting paragraph to the results section to briefly introduce the topic, your goals and hypothesis and a short summary of what you did in this work. Many readers start reading the manus from the results and it should be understandable.

**Response:** Thank you very much for the suggestion.

**Comment 8:** All the abbreviations should be explained when used the first time in the manuscript. In addition, if you can avoid any of the abbreviations, it is preferred to write only full text.

**Response:** Thank you very much for the suggestion, revised accordingly.

**Comment 9:** Your conclusion is too long. Please cut to to less than 100 words and in one paragraph. You can move some part of it to the discussions if you prefer so.

**Response:** Thank you very much for the suggestion, revised: Fungicide application strongly interference on soil microbial community, remarkably decreased bacterial and fungal communities’ diversity, the respiration of bacteria is more sensitive and approximately three times than fungi. The abundance of dominant bacterial *Proteobacteria* decreased and fungal *Ascomycota* increased in fungicide treated soil, especially enriched in beneficial microorganisms and reduced in pathogen, including bacterial of *Bacillus*, *Pseudomonas* and *Arthrobacter*, fungi of *Fusarium*, *Mortierella* and *Botrytis*. The adaptability and enrichment of beneficial microorganisms and reduction of pathogenic bacteria in soil may be the role of fungicides application.
